# Use of mouse-tracking software to detect faking-good behavior on personality questionnaires: an explorative study

**DOI:** 10.1038/s41598-020-61636-5

**Published:** 2020-03-16

**Authors:** Cristina Mazza, Merylin Monaro, Franco Burla, Marco Colasanti, Graziella Orrù, Stefano Ferracuti, Paolo Roma

**Affiliations:** 1 Department of Human Neuroscience, Sapienza University of Rome, Rome, Italy; 2 Department of General Psychology, University of Padova, Padova, Italy; 3 Department of Surgical, Medical, Molecular & Critical Area Pathology, University of Pisa, Pisa, Italy

**Keywords:** Cognitive control, Decision, Human behaviour

## Abstract

The aim of the present study was to explore whether kinematic indicators could improve the detection of subjects demonstrating faking-good behaviour when responding to personality questionnaires. One hundred and twenty volunteers were randomly assigned to one of four experimental groups (honest unspeeded, faking-good unspeeded, honest speeded, and faking-good speeded). Participants were asked to respond to the MMPI-2 underreporting scales (L, K, S) and the PPI-R Virtuous Responding (VR) scale using a computer mouse. The collected data included T-point scores on the L, K, S, and VR scales; response times on these scales; and several temporal and spatial mouse parameters. These data were used to investigate the presence of significant differences between the two manipulated variables (honest vs. faking-good; speeded vs. unspeeded). The results demonstrated that T-scores were significantly higher in the faking-good condition relative to the honest condition; however, faking-good and honest respondents showed no statistically significant differences between the speeded and unspeeded conditions. Concerning temporal and spatial kinematic parameters, we observed mixed results for different scales and further investigations are required. The most consistent finding, albeit with small observed effects, regards the L scale, in which faking-good respondents took longer to respond to stimuli and outlined wider mouse trajectories to arrive at the given response.

## Introduction

One of the main limitations of the use of self-report questionnaires to assess personality is that such tests are vulnerable to faking behavior^1^—that is, the tendency to deliberately distort one’s responses in order to fulfil personal goals^2^. In one form of faking, respondents exaggerate or create symptoms to emphasize their psychological suffering and discomfort (faking-bad); in another, respondents present themselves in a particularly favourable fashion, emphasizing their desirable traits and rejecting their undesirable ones (faking-good). Faking behaviour is widespread in many contexts, with alarming estimates of prevalence (e.g., 30–50% in personnel selection^3^ and up to 30% in forensic settings^4,5^). Many studies have focused on faking-bad behaviour and developed tools to facilitate its detection; such tools include the Structured Interview of Reported Symptoms-2 (SIRS-2)^6^, the Structured Inventory of Malingered Symptomatology (SIMS)^7^, and the Inventory of Problems-29^8^. Faking-bad behaviour has received more research attention^9,10^ perhaps because its welfare/social costs (in terms of, e.g. insurance compensation) are more easily recognizable; thus, the literature on the subject is not as rich and instruments to identify faking-good behaviour are lacking; for this reason, the present study focused on faking-good behavior, specifically.

Analysis of validity scales is one of the most commonly used methods to detect fakers. Validity scales were designed to gather information on the validity and interpretability of self-report questionnaires by exploring the presence of what Paulhus defined as “responding bias”^11^. Responding bias is the systematic tendency to answer items of a self-report test in a way that interferes with accurate self-presentation; to some extent, this bias may be linked to intentional distortions of one’s self-image (i.e., faking). Starting from the observation that the main limitation of self-report questionnaires and their corresponding validity scales is the transparency of their items—which may render the measured construct evident to test-takers—research has focused on exploring possible indirect methods of detecting faking respondents.

Since the 1970s, research has studied the application of response time (RT) for detecting fakers^12^, yielding encouraging results. Specifically, the self-schema model^13^ suggests that fakers, while answering a self-report questionnaire, will take longer to respond to items than honest subjects. This result was empirically confirmed by a meta-analysis conducted in 2016 by Maricuțoiu and Sârbescu^14^, who concluded that lying takes more time because it is either more cognitively demanding than telling the truth^15–18^ or because it causes higher levels of arousal due to fear of detection [*d* = 0.23; 95%C.I. 0.07, 0.39]^19^. The meta-analysis also highlighted that the difference in RTs between honest respondents and fakers is only significant when items are endorsed, not rejected.

Recent research has indicated^20,21^ that speeded tests (i.e., those that limit the time available to respondents, instructing them to answer as quickly as possible) increase faking behaviour, making it more readily detectable. To explain these findings, Shalvi *et al*.^20^ suggested that subjects with limited time to reflect lie more frequently, whereas those with more time available choose their answers more cautiously in an attempt to moderate their faking behaviour. This idea was confirmed in a recent study^17,22^, in which time pressure led fakers to significantly improve their self-presentation on the L-r and K-r scales of the MMPI-2-RF (η^2^_p_ = 0.243). Roma *et al*.^23^ also pointed out that time pressure can be useful in distinguishing between honest respondents and fakers, even when items are rejected: in their research, no difference in RTs between honest respondents and fakers was registered in the unspeeded condition, while a statistically significant difference was observed in the speeded condition (MMPI-2 L scale η^2^ = 0.481; MMPI-2 K scale η^2^ = 0.457; MMPI-2 S scale η^2^ = 0.011). More recently, Verschuere, Köbis, Bereby-Meyer, Rand, and Shalvi (2018)^24^ carried out a meta-analysis of 21 studies, finding that, when RT is considered exclusively, honesty requires less time than faking; however, when cognitive load is also considered (e.g., via time pressure, ego depletion, stress, sleep deprivation, or use of a foreign language), RT may be less able to distinguish between honest respondents and fakers, because the cognitive load may hinder honest respondents’ ability to quickly tell the truth [*g* = −0.184; 95%C.I. −0.35, −0.02].

Recently, a new and promising technique has been implemented in faking detection research: mouse tracking. Mouse tracking is a procedure that enables researchers to trace mouse trajectories by recording the cursor’s position 60–75 times per second^25^. It is thought that mouse trajectories can be used to explore the real-time evolution of mental processes in the execution of decision-making tasks (e.g., personality questionnaires), since motor movements are continuously adjusted to underlying cognitive processes^26–31^. When choosing between two dichotomous alternatives, an individual must solve the underlying cognitive conflict^32^. Recently, Freeman and Ambady developed a software, MouseTracker^25^, to track and analyse mouse trajectories during the execution of tasks requiring a choice between multiple alternatives. This software gathers information about not only the real-time positions of the cursor, but also the time elapsed between two consecutive mouse movements. This enables the software to determine the speed and acceleration of every trajectory, calculate the average path of each participant, and compare each path to those of other subjects on the same task. The software also assesses two parameters: maximum deviation (MD), which represents the largest perpendicular deviation of the actual trajectory from the idealized one; and area under the curve (tAUC), which is the geometric area between the two trajectories. Research has found that rejecting an item is more cognitively demanding than endorsing an item; thus, the former engenders a longer RT^29^. Although RTs recorded by mouse tracking do not necessarily overlap with the RTs registered in the aforementioned studies, the mouse tracking technique has nonetheless proven useful for lie detection^33^. Previous studies^33–35^ have shown that, when half of a sample answered an autobiographical questionnaire truthfully and the other half answered according to fake profiles that had been learned just prior to testing, honest participants followed the more direct trajectory to the desired answer while fakers showed trajectories that initially converged towards the actual autobiographical information and then switched to the opposite direction to select the relevant alternative.

The present study aimed at generating insight into the relationship between different approaches to identifying faking-good behaviour on the underreporting validity scales of two widely used personality questionnaires: the L, K, and S underreporting scales of the MMPI-2 and the Virtuous Responding (VR) scale of the PPI-R. These scales were chosen because they were designed to detect the acknowledgment of uncommon virtues and the tendency to omit negative features of personality in order to present oneself in a better light. Specifically, the analysis considered T-scores (calculated with respect to normative data provided in the technical manual), RTs, and mouse trajectories; it also measured the impact of time pressure on each method.

The hypotheses were as follows:

H1) T-scores on the underreporting scales (L, K, S) of the MMPI-2 and the VR scale of the PPI-R would be higher in the faking-good condition compared to the honest one.

H2) T-scores on the underreporting scales (L, K, S) of the MMPI-2 and the VR scale of the PPI-R would be higher in the faking-good speeded condition compared to the faking-good unspeeded condition; T-scores of honest respondents would not show any significant difference between the speeded and unspeeded conditions.

H3) Mouse movements would be faster in the speeded condition relative to the unspeeded condition.

H4) Mouse movements would be slower in the faking-good condition relative to the honest condition.

H5) Faking-good respondents’ mouse trajectories would be wider than those of honest respondents.

H6) Mouse trajectories for subjects in the speeded condition would be wider than those in the unspeeded condition.

## Materials and Methods

### Participants

The sample was comprised of 120 young adults who voluntarily participated in the study. The only prerequisite for taking part in the research was the ability to read questions on a computer monitor, understand their meaning, and subsequently answer via a mouse. To limit noisy variables, only Caucasian female subjects aged 18 to 30 years (M = 22.45; SD = 3.13) who were non-psychology graduates (i.e., they had not studied in a psychology faculty) were recruited. Participants were randomly assigned to one of four experimental conditions, defined by a combination of the two manipulated variables relating to the instructions (honest [H] vs. faking-good [FG]) and time pressure (speeded [S] vs. unspeeded [U]). Group 1 (N = 30) (M_age_ = 22.43; SD = 2.79) was an honest–faking-good unspeeded (H-FG/U) group; group 2 (N = 30) (M_age_ = 23.80; SD = 2.79) was a faking-good–honest unspeeded (FG-H/U) group; group 3 (N = 30) (M_age_ = 21.13; SD = 2.35) was an honest–faking-good speeded (H-FG/S) group, and group 4 (N = 30) (M_age_ = 22.43; SD = 3.27) was a faking-good–honest speeded (FG-H/S) group. It has been calculated that a sample size of 30 is sufficiently large to achieve statistical power (1-β) = 0.8, given a significance level (α) of 0.05 and a medium effect size (d) of 0.5^36^. Group 3 was younger, on average, than all other groups [F_(3,116)_ = 3.889, *p* = 0.011].

Between September and October 2019, an additional 120 young adult volunteers were recruited as an out-of-sample evaluation group for the model built on the original sample. Participants were recruited in the same way as the prior sample and met the same inclusion/exclusion criteria. They received no reward for their participation. All subjects were aged 18 to 29 years old (M = 22.73; SD = 2.84); half were male and the other half were female; all were Caucasian. Participants were randomly assigned to one of four experimental groups, following the same manipulation of factors, instructions (honest vs. faking-good), and time pressure (speeded vs. unspeeded) as the original sample. Group 1 (N = 30) (M_age_ = 23.53; SD = 2.70) was an honest–faking-good unspeeded (H/FG-U) group; group 2 (N = 30) (M_age_ = 21.97; SD = 2.57) was a faking-good–honest unspeeded (FG/H-U) group; group 3 (N = 30) (M_age_ = 22.67; SD = 2.91) was an honest–faking-good speeded (H/FG-S) group; and group 4 (N = 30) (M_age_ = 22.77; SD = 3.08) was a faking-good–honest speeded (FG/H-S) group. No statistically significant differences were observed between groups with respect to age. As the statistical analyses on the original sample (see “Results” section) highlighted that honest and faking-good respondents mainly differed in their responses on the L scale, only, the out-of-sample group were only administered items on this scale. Moreover, in the second data collection, two methodological shortcomings were fixed: i) the instructions given to honest and faking-good groups were matched, so that honest were informed that the test contained features designed to detect faking; and ii) the position of the response labels (true vs. false) was inverted to eliminate possible response biases due to the allocation of these labels.

All participants provided informed consent before the research began. They did not receive any compensation for their participation. The experimental procedure was approved by the local ethics committee (Board of the Department of Human Neuroscience, Faculty of Medicine and Dentistry, Sapienza University of Rome), in accordance with the Declaration of Helsinki.

### Materials

#### Underreporting validity scales (L, K, S) of the MMPI-2

The Minnesota Multiphasic Personality Inventory-2 (MMPI-2)^37^ is a 51-scale self-report questionnaire that is used to measure personality and psychopathology. It is comprised of 567 items that each require a dichotomous answer (true vs. false). The MMPI-2 is largely used in forensic and evaluation settings^38–42^. The present study used three MMPI-2 validity scales: Lie (L), Correction (K), and Superlative Self-Presentation (S). The L scale, composed of 15 items, was designed to detect the acknowledgment of uncommon virtues and the tendency to offer a more socially acceptable image of oneself (e.g., “I do not always tell the truth”). Most of the items in this scale require respondents to choose “false” in order to answer in a socially desirable way. The K scale, composed of 30 items, was designed to detect defensiveness in a more subtle way, investigating respondents’ adjustment and emotional control (e.g., “criticism or scolding hurts me terribly”). The S scale, composed of 50 items, was designed to identify self-presentation as highly virtuous and extremely well adjusted in any context (e.g., “I have never felt better in my life than I do now”). The higher a respondent scores on these scales, the higher the chance that he or she is presenting an overly positive self-image. The Italian version of the MMPI-2 was edited by Pancheri and Sirigatti^43,44^.

#### Virtuous responding (VR) validity scale of the PPI-R

The Psychopathic Personality Inventory-Revised (PPI-R)^45^ is a 154-item personality questionnaire, articulated within 8 subscales, that assesses traits associated with psychopathy. Respondents must answer each item on a 4-point scale (true vs. true enough vs. false enough vs. false). The present study used the PPI-R Virtuous Responding (VR) validity scale, which is composed of 13 items (e.g., “I’ve never desired to hurt someone”) and was designed to detect underreporting. The Italian version of the PPI-R was edited by La Marca *et al*. (2008)^46^.

#### Research design

A mixed design was implemented. The two manipulated factors were instructions (H vs. FG) and time pressure (U vs. S). As described above, participants were randomly assigned to one of four experimental groups: H-FG/U, FG-H/U, H-FG/S, and FG-H/S.

In the first group (H-FG/U), subjects completed the tests (the L, K, and S scales of the MMPI-2; and the VR scale of the PPI-R) without time pressure, first with the instruction to respond honestly (1a) and then with the instruction to fake good (1b). Specifically, the instructions were as follows:

1a) We are interested in some characteristics of your personality. We want you to take this test in a totally sincere fashion. After reading each item you should take all the time you need to respond in the best way.

1b) You just completed the test honestly. Now imagine that you are applying for a desired job. In this situation, it would be to your advantage to appear as if you were completely normal and psychologically healthy. Stated differently, we want you to take this test and deliberately fake good. Pay attention, because the questionnaire contains features designed to detect faking, and your intent is to respond in a way that your deception cannot be detected. After reading each item you should take all the time you need to respond in the best way, according to this instruction.

In the second group (FG-H/U), subjects completed the test without time pressure, first with the instruction to fake good (2a) and then with the instruction to respond honestly (2b). Specifically, the instructions were as follows:

2a) We are interested in some characteristics of your personality. Imagine you are applying for a desired job. In this situation, it would be to your advantage to appear as if you were completely normal and psychologically healthy. Stated differently, we want you to take this test and deliberately fake good. Pay attention, because the questionnaire contains features designed to detect faking, and your intent is to respond in a way that your deception cannot be detected. After reading each item you should take all the time you need to respond in the best way, according to this instruction.

2b) You just completed the test dishonestly. Now, we are interested in some real characteristics of your personality. We want you to take this test in a totally sincere fashion. After reading each item you should take all the time you need to respond in the best way.

In the third group (H-FG/S), subjects completed the test with time pressure, first with the instruction to respond honestly (3a) and then with the instruction to fake good (3b). Specifically, the instructions were as follows:

3a) We are interested in some characteristics of your personality. We want you to take this test in a totally honest fashion. After reading each item you should respond as quickly as possible.

3b) You just completed the test honestly. Now imagine that you are applying for a desired job. In this situation it would be to your advantage to appear as if you were completely normal and psychologically healthy. Stated differently, we want you to take this test and deliberately fake good. Pay attention, because the questionnaire contains features designed to detect faking, and your intent is to respond in a way that your deception cannot be detected. After reading each item you should respond as quickly as possible. Short response time is an important factor in this test.

Finally, in the fourth group (FG-H/S), subjects completed the test with time pressure, first with the instruction to fake good (4a) and then with the instruction to respond honestly (4b). Specifically, the instructions were as follows:

4a) We are interested in some characteristics of your personality. Imagine you are applying for a desired job. In this situation it would be to your advantage to appear as if you were completely normal and psychologically healthy. Stated differently, we want you to take this test and deliberately fake good. Pay attention, because the questionnaire contains features designed to detect faking, and your intent is to respond in a way that your deception cannot be detected. After reading each item you should respond as quickly as possible. Short response time is an important factor in this test.

4b) You just completed the test dishonestly. Now, we are interested in some real characteristics of your personality. We want you to take this test in a totally honest fashion. After reading each item you should respond as quickly as possible. Short response time is an important factor in this test.

#### Procedure and stimuli

The experimental task was completed individually in a neutral, quiet room in the Human Neuroscience Department of Sapienza, University of Rome. Subjects, placed approximately 60 cm from the screen, completed the test on a 15-inch display laptop with a Microsoft Windows operating system. After the initial reception, participants went through the following procedure: a) completion of a consent form, b) completion of a demographic questionnaire, c) assignment to one of the four experimental groups previously described, d) completion of the experimental task (scripts L, K, S, and VR) with their respective group’s first instructions (the abovementioned instructions 1a, 2a, 3a, and 4a), e) projection of an unrelated short video, and f) completion of the experimental task (scripts L, K, S, and VR) with their respective group’s second instructions (the abovementioned instructions 1b, 2b, 3b, and 4b) (see Table 1).

**Table 1:** Experimental conditions (i.e., combination of two factors).

1st completion	Unspeeded	Speeded
Honest	Group 1	Group 3
Faking-good	Group 2	Group 4
**2nd completion**	**Unspeeded**	**Speeded**
Honest	Group 2	Group 4
Faking-good	Group 1	Group 3

**Figure 1:**
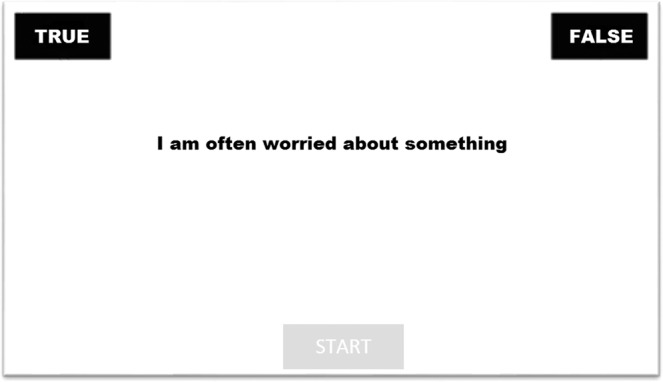
Example of an experimental trial, as seen by the participant. Each question appeared after the participant clicked the START button.

The experimental task consisted of the 96 stimuli (i.e., items) belonging to the underreporting scales (L, K, S) of the MMPI-2 and the VR scale of the PPI-R (see Table S1). The presentation order of the stimuli reflected the item appearance order in the MMPI-2 protocol, followed by the item appearance order of the VR scale in the PPI-R. Stimuli were presented in the central display of the computer screen. Participants had to initiate the presentation of each question by clicking (with the mouse) a START button located in the centre-lower part of the screen. For items relating to the MMPI-2 validity scales, participants were asked to respond to each question by clicking (with the mouse) one of two alternative response buttons (TRUE vs. FALSE) presented in the upper part of the computer screen: one in the upper-left corner and one in the upper-right corner (see Fig. 1). For items relating to the VR scale of the PPI-R, participants had to choose one of four alternative response buttons (TRUE vs. TRUE ENOUGH vs. FALSE ENOUGH vs. FALSE).

#### Collected measures

During the experimental task, the MouseTracker software^25^ automatically recorded a number of features relating to the response of the mouse in spatial and temporal terms. Mouse parameters that the literature reported to be the most sensitive to deception detection were collected^5,33,35,47^. Specifically, the following features were captured for each mouse trajectory:

Spatial features

- Maximum deviation (MD): maximum perpendicular distance between the actual and idealized trajectories
- Area under the curve (tAUC): geometric area between the actual and idealized trajectories

Temporal features

- Reaction time (RT): time between the presentation of the question and the click on the response button
- Maximum deviation time (MD-time): time taken to reach the point of maximum deviation
- Velocity over time on x-axis (vel_x_): velocity of the mouse along the x-axis between two-time frames
- Velocity over time on y-axis (vel_y_): velocity of the mouse along the y-axis between two-time frames

The idealized trajectory represented the virtual straight line connecting the starting point to the endpoint (the response label). For example, if the start button (placed in the centre-lower part of the screen) and the response labels (placed in the upper-left and upper-right corners) formed a triangle, the idealized response trajectory would correspond to the side of the triangle that connected the START button to each response label. Because the recorded trajectories had different lengths, each motor response was time normalized in order to permit trials to be averaged and compared. Using linear interpolation, the software calculated time normalization in 101 temporal frames. As a result, each trajectory had 101 temporal frames and each time frame had corresponding X and Y coordinates^25^. Finally, for each spatial (MD, tAUC) and temporal (RT, MD-time, vel_x_, vel_y_) parameter, the average response value on each scale (L, K, S, VR) was computed, generating 24 variables (see Table S2). The T-scores on the underreporting scales (L, K, S) of the MMPI-2 and the VR scale of the PPI-R were computed. The term T scores is used to denote “test scores that -within rounding errors- have a mean of 50 and a standard deviation of 10 in the normal group”^48^. It is calculated by using the following linear transformation: T = 50 + 10 (Xi-x)/s in which Xi is the raw score to be converted, x is the mean, and s is the standard deviation of the norm group^49^.

All measures, conditions, data exclusions, and methods used to determine the sample sizes are reported here.

### Univariate statistical analysis

Mixed ANOVA models were used to test the six hypotheses (H1–H6) on the original sample of 120 participants. In more detail, comparisons were drawn between the performances obtained by the four experimental groups on each scale (L, K, S, VR), in terms of T-scores (H1 and H2), temporal features (H3 and H4), and spatial features (H5 and H6). Means and standard deviations of all dependent variables are shown in Table S3. The effect sizes of the score differences between groups were recorded; with respect to magnitude, η_G_^2^ = 0.02 was considered indicative of a small effect, η_G_^2^ = 0.13 a medium effect, and η_G_^2^ = 0.26 a large effect^50^. All analyses were performed using the “ez” package in the R software^51^.

### H1 and H2

A mixed ANOVA was computed on the T-scores of each scale (L, K, S, VR), and the results demonstrated a significant effect of instructions on each scale. In other words, faking-good respondents obtained significantly higher T-scores on the L, K, S, and VR scales relative to honest respondents. Table 2 reports the ANOVA outputs that highlight the statistically significant results.

**Table 2:** Significant results from the ANOVA mixed models computed on the T-scores of the L, K, and S scales of the MMPI-2 and the VR scale of the PPI-R.

T-score variable	Effect	F	*p*-value	η_G_^2^	95% CI
T-score S scale	Instructions	F_(1,118)_ = 222.12	6.676e^−29^	0.34 (large)	[0.21, 0.48]
T-score K scale	Instructions	F_(1,118)_ = 152.63	5.219e^−23^	0.30 (large)	[0.16, 0.43]
T-score L scale	Instructions	F_(1,118)_ = 146.55	2.011e^−22^	0.32 (large)	[0.18, 0.46]
T-score VR scale	Instructions	F_(1,118)_ = 162.30	6.492e^−24^	0.33 (large)	[0.19, 0.46]

F-score, *p*-value, and effect size (η_G_^2^) are reported for each significant effect. With respect to magnitude, η_G_^2^ = 0.02 is considered indicative of a small effect, η_G_^2^ = 0.13 a medium effect, and η_G_^2^ = 0.26 a large effect^50^.

Furthermore, the ANOVAs indicated that there was no significant effect of time pressure on any scale (T-score S scale: F_(1,118)_ = 0.19, *p* = 0.664, η_G_^2^ < 0.02; T-score K scale: F_(1,118)_ = 0.21, *p* = 0.649, η_G_^2^ < 0.02; T-score L scale: F_(1,118)_ = 0.96, *p* = 0.328, η_G_^2^ < 0.02; T-score VR scale: F_(1,118)_ = 0.39, *p* = 0.531, η_G_^2^ < 0.02). Simply put, both faking-good and honest respondents showed no statistically significant differences in T-scores between the speeded and unspeeded conditions. Similarly, no statistically significant results were generated by the interaction between time pressure and instructions (T-score S scale: F_(1,118)_ = 0.03, *p* = 0.873, η_G_^2^ < 0.02; T-score K scale: F_(1,118)_ = 0.22, *p* = 0.639, η_G_^2^ < 0.02; T-score L scale: F_(1,118)_ = 1.64, *p* = 0.20, η_G_^2^ < 0.020; T-score VR scale: F_(1,118)_ = 0.03, *p* = 0.857, η_G_^2^ < 0.02).

### H3 and H4

Mouse movements were temporally described by four kinematic features: RT, MD-time, vel_x_, and vel_y_. For each feature, a mixed ANOVA was run to compare the temporal responses of the four experimental groups on the L, K, S, and VR scales. To resolve the multiple testing problem, the Bonferroni correction was applied, dividing the *p*-value by the number of tested features and setting the significance level to 0.0125^52^. Table 3 reports the output of the temporal features that showed statistically significant effects. It is worth noting that there was a main effect of time pressure on RT and MD-time on all scales, except for MD-time on the VR scale (MD-time VR scale: F_(1,118)_ = 4.78, *p* = 0.03, η_G_^2^ = 0.03). These results highlight that participants in the speeded condition were faster in responding than participants in the unspeeded condition; the former showed smaller RTs and took less time to reach the point of maximum deviation (MD-time). Conversely, the analyses did not reveal any significant effect of time pressure on vel_x_ or vel_y_ in any scale (vel_x_ S scale: F_(1,118)_ = 0.000004, *p* = 0.998, η_G_^2^ < 0.02; vel_x_ K scale: F_(1,118)_ = 0.21, *p* = 0.646, η_G_^2^ < 0.02; vel_x_ L scale: F_(1,118)_ = 0.73, *p* = 0.395, η_G_^2^ < 0.02; vel_x_ VR scale: F_(1,118)_ = 1.04, *p* = 0.310, η_G_^2^ < 0.02; vel_y_ S scale: F_(1,118)_ = 0.45, *p* = 0.504, η_G_^2^ < 0.02; vel_y_ K scale: F_(1,118)_ = 1.32, *p* = 0.252, η_G_^2^ < 0.02; vel_y_ L scale: F_(1,118)_ = 0.16, *p* = 0.688, η_G_^2^ < 0.02; vel_y_ VR scale: F_(1,118)_ = 1.38, *p* = 0.242, η_G_^2^ < 0.02).

**Table 3:** Significant results from the ANOVA mixed models computed on RT, MD-time, vel_x_, and vel_y_ for each scale (L, K, S, VR). F-score, *p*-value, and effect size (η_G_^2^) are reported for each significant effect.

Temporal variable	Effect	F	*p*-value	η_G_^2^	95% CI
RT S scale	Time pressure	F_(1,118)_ = 18.58	3.395e^−05^	0.09 (small)	[0.01, 0.18]
RT K scale	Time pressure	F_(1,118)_ = 19.04	2.753e^−05^	0.10 (small)	[0.01, 0.18]
RT L scale	Time pressure	F_(1,118)_ = 23.11	4.559e^−06^	0.12 (small)	[0.02, 0.20]
RT VR scale	Time pressure	F_(1,118)_ = 10.36	1.661e^−03^	0.06 (small)	[0.00, 0.14]
MD-time S scale	Time pressure	F_(1,118)_ = 18.78	3.097e^−05^	0.09 (small)	[0.01, 0.18]
MD-time K scale	Time pressure	F_(1,118)_ = 20.60	1.374e^−05^	0.11 (small)	[0.01, 0.19]
MD-time L scale	Time pressure	F_(1,118)_ = 19.27	2.481e^−05^	0.09 (small)	[0.01, 0.19]
RT L scale	Instructions	F_(1,118)_ = 17.30	6.096e^−05^	0.05 (small)	[0.00, 0.14]
MD-time L scale	Instructions	F_(1,118)_ = 9.21	2.962e^−03^	0.03 (small)	[0.00, 0.12]
velx S scale	Instructions	F_(1,118)_ = 191.33	1.878e^−26^	0.28 (large)	[0.15, 0.42]
velx K scale	Instructions	F_(1,118)_ = 140.99	7.097e^−22^	0.27 (large)	[0.14, 0.41]
velx L scale	Instructions	F_(1,118)_ = 151.25	7.069e^−23^	0.32 (large)	[0.18, 0.46]
vely K scale	Instructions	F_(1,118)_ = 6.76	1.050e^−02^	<0.02	[0.00, 0.08]
vely VR scale	Instructions	F_(1,118)_ = 9.26	0.003	0.02 (small)	[0.00, 0.10]

The *p*-value is set to 0.0125, according to the Bonferroni correction. With respect to magnitude, η_G_^2^ = 0.02 is considered indicative of a small effect, η_G_^2^ = 0.13 a medium effect, and η_G_^2^ = 0.26 a large effect^50^.

**Figure 2:**
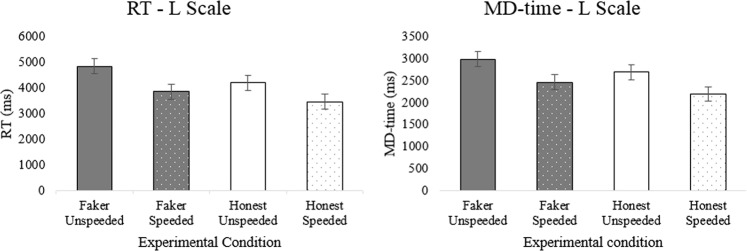
Bar plots representing the performance of the four experimental groups in terms of RT (left plot) and MD-time (right plot) on the MMPI-2 L scale.

Regarding the effect of instructions, faking-good respondents were significantly slower than honest respondents in terms of RT and MD-time only on the L scale (see Fig. 2). For all other scales (S, K, VR), there was no main effect of instructions on RT and MD-time (RT S scale: F_(1,118)_ = 1.85, *p* = 0.176, η_G_^2^ < 0.02; RT K scale: F_(1,118)_ = 3.78, *p* = 0.054, η_G_^2^ < 0.02; RT VR scale: F_(1,118)_ = 1.02, *p* = 0.314, η_G_^2^ < 0.02; MD-time S scale: F_(1,118)_ = 1.42, *p* = 0.236, η_G_^2^ < 0.02; MD-time K scale: F_(1,118)_ = 3.59, *p* = 0.060, η_G_^2^ < 0.02; MD-time VR scale: F_(1,118)_ = 1.36, *p* = 0.245, η_G_^2^ < 0.02). Differently, there was a main effect of instructions on vel_x_ for all scales, except for VR. Moreover, there was an effect of instructions on vel_y_ for the K and VR scales, but not on the S and L scales (vel_y_ S scale: F_(1,118)_ = 2.24, *p* = 0.137, η_G_^2^ < 0.02; vel_y_ L scale: F_(1,118)_ = 2.61, *p* = 0.109, η_G_^2^ < 0.02). This means that participants in the honest condition were faster than faking-good respondents in moving along the x-axis (vel_x_) when responding to items on the L, K, and S scales. They were also faster than faking-good respondents in moving along the y-axis (vel_y_) when responding to items on the K and VR scales.

Finally, the interaction between time pressure and instructions did not show any statistically significant results for any of the considered variables and scales (RT S scale: F_(1,118)_ = 0.13, *p* = 0.715, η_G_^2^ < 0.02; RT K scale: F_(1,118)_ = 0.06, *p* = 0.802, η_G_^2^ < 0.02; RT L scale: F_(1,118)_ = 0.95, *p* = 0.333, η_G_^2^ < 0.02; RT VR scale: F_(1,118)_ = 0.05, *p* = 0.823, η_G_^2^ < 0.02; MD-time S scale: F_(1,118)_ = 0.12, *p* = 0.729, η_G_^2^ < 0.02; MD-time K scale: F_(1,118)_ = 0.07, *p* = 0.788, η_G_^2^ < 0.02; MD-time L scale: F_(1,118)_ = 0.01, *p* = 0.902, η_G_^2^ < 0.02; MD-time VR scale: F_(1,118)_ = 0.07, *p* = 0.794, η_G_^2^ < 0.02; vel_x_ S scale: F_(1,118)_ = 0.09, *p* = 0.753, η_G_^2^ < 0.02; vel_x_ K scale: F_(1,118)_ = 0.41, *p* = 0.53, η_G_^2^ < 0.02; vel_x_ L scale: F_(1,118)_ = 1.41, *p* = 0.24, η_G_^2^ < 0.02; vel_x_ VR scale: F_(1,118)_ = 0.41, *p* = 0.525, η_G_^2^ < 0.02; vel_y_ S scale: F_(1,118)_ = 0.12, *p* = 0.734, η_G_^2^ < 0.02; vel_y_ K scale: F_(1,118)_ = 0.23, *p* = 0.629, η_G_^2^ < 0.02; vel_y_ L scale: F_(1,118)_ = 1.53, *p* = 0.219, η_G_^2^ < 0.02; vel_y_ VR scale: F_(1,118)_ = 0.14, *p* = 0.706, η_G_^2^ < 0.02).

### H5 and H6

The shape of the mouse trajectories was described by two spatial features: MD and tAUC. Similar to the analysis of temporal features, a mixed ANOVA was run to compare the mouse trajectories of the four experimental groups on the L, K, S, and VR scales. After the Bonferroni correction, the significance level was set to 0.025. The significant outputs are reported in Table 4.

**Table 4:** Significant results from the ANOVA mixed models computed on MD and tAUC for each scale (L, K, S, VR). F-score, *p*-value and effect size (η_G_^2^) are reported for each significant effect.

Spatial variable	Effect	F	*p*-value	η_G_^2^	95% CI
MD S scale	Time pressure	F_(1,118)_ = 6.62	1.130e^−02^	0.04 (small)	[0.00, 0.11]
MD K scale	Time pressure	F_(1,118)_ = 5.15	2.506e^−02^	0.03 (small)	[0.00, 0.10]
MD L scale	Time pressure	F_(1,118)_ = 8.72	3.792e^−03^	0.05 (small)	[0.00, 0.13]
MD L scale	Instructions	F_(1,118)_ = 6.15	1.451e^−02^	<0.02	[0.00, 0.08]
tAUC L scale	Instructions	F_(1,118)_ = 5.43	2.146e^−02^	<0.02	[0.00, 0.08]

The *p*-value is set to 0.025, according to the Bonferroni correction. With respect to magnitude, η_G_^2^ = 0.02 is considered indicative of a small effect, η_G_^2^ = 0.13 a medium effect, and η_G_^2^ = 0.26 a large effect^50^.

**Figure 3:**
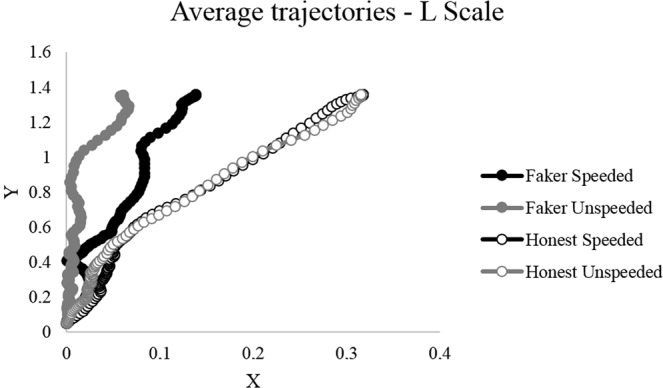
Average trajectories of the subjects of each experimental group when responding to items of the L scale. To enable a direct comparison of the four conditions, all trajectories were horizontally remapped.

The results demonstrate a main effect of instructions on MD and tAUC for the L scale, only. In other words, faking-good respondents had wider trajectories than honest respondents on the L scale (see Fig. 3), regardless of the time pressure condition. For all other scales (S, K, VR), there was no significant effect of instructions on MD and tAUC (MD S scale: F_(1,118)_ = 1.81, *p* = 0.181, η_G_^2^ < 0.02; MD K scale: F_(1,118)_ = 3.91, *p* = 0.052, η_G_^2^ < 0.02; MD VR scale: F_(1,118)_ = 1.07, *p* = 0.302, η_G_^2^ < 0.02; tAUC S scale: F_(1,118)_ = 2.42, *p* = 0.122, η_G_^2^ < 0.02; tAUC K scale: F_(1,118)_ = 2.19, *p* = 0.142, η_G_^2^ < 0.02; tAUC VR scale: F_(1,118)_ = 1.18, *p* = 0.279, η_G_^2^ < 0.02).

Regarding the effect of time pressure, the MD parameter revealed that the mouse trajectories were wider for subjects in the speeded condition compared to those in the unpressured condition for all scales except for the VR scale (MD VR scale: F_(1,118)_ = 1.01, *p* = 0.317, η_G_^2^ < 0.02). However, there was no main effect of time pressure on tAUC (tAUC S scale: F_(1,118)_ = 3.85, *p* = 0.052, η_G_^2^ = 0.03; tAUC K scale: F_(1,118)_ = 2.27, *p* = 0.134, η_G_^2^ < 0.02; tAUC L scale: F_(1,118)_ = 3.12, *p* = 0.080, η_G_^2^ < 0.02; tAUC VR scale: F_(1,118)_ = 1.53, *p* = 0.219, η_G_^2^ < 0.02).

The interaction between time pressure and instructions showed no statistically significant results for any of the two spatial features (MD S scale: F_(1,118)_ = 0.41, *p* = 0.523, η_G_^2^ < 0.02; MD K scale: F_(1,118)_ = 0.01, *p* = 0.917, η_G_^2^ < 0.02; MD L scale: F_(1,118)_ = 2.60, *p* = 0.110, η_G_^2^ < 0.02; MD VR scale: F_(1,118)_ = 0.25, *p* = 0.621, η_G_^2^ < 0.02; tAUC S scale: F_(1,118)_ = 0.96, *p* = 0.328, η_G_^2^ < 0.02; tAUC K scale: F_(1,118)_ = 0.00002, *p* = 0.996, η_G_^2^ < 0.02; tAUC L scale: F_(1,118)_ = 0.58, *p* = 0.450, η_G_^2^ < 0.02; tAUC VR scale: F_(1,118)_ = 0.003, *p* = 0.956, η_G_^2^ < 0.02).

Table 5 shows significant results from the ANOVA mixed models computed on the T-scores, RT, MD-time, vel_x_, vel_y_, MD and tAUC of the L scale of the MMPI-2 for the 120 additional volunteers recruited as an out-of-sample evaluation group.

**Table 5:** Significant results from the ANOVA mixed models computed on the T-scores, RT, MD-time, vel_x_, vel_y_, MD and tAUC of the L scale of the MMPI-2 for the 120 additional volunteers recruited as an out-of-sample evaluation group, p-value, and effect size (η_G_^2^) are reported for each significant effect.

L scale variables	Effect	F	*p*-value	η_G_^2^	95% CI
RT L scale	Time pressure	F_(1,118)_ = 26.82	9.298e^−07^	0.15 (medium)	[0.02, 0.22]
MD-time L scale	Time pressure	F_(1,118)_ = 22.79	5.223e^−06^	0.12 (small)	[0.02, 0.20]
MD L scale	Time pressure	F_(1,118)_ = 5.12	2.542e^−02^	0.03 (small)	[0.00, 0.10]
T-score L scale	Instructions	F_(1,118)_ = 320.98	1.856e^−35^	0.44 (large)	[0.30, 0.57]
RT L scale	Instructions	F_(1,118)_ = 24.55	2.441e^−06^	0.05 (small)	[0.00, 0.14]
MD-time L scale	Instructions	F_(1,118)_ = 8.11	5.200e^−03^	0.02 (small)	[0.00, 0.10]
MD L scale	Instructions	F_(1,118)_ = 20.18	1.657e^−05^	0.04 (small)	[0.00, 0.13]
tAUC L scale	Instructions	F_(1,118)_ = 12.38	6.170e^−04^	0.02 (small)	[0.00, 0.10]
velx L scale	Instructions	F_(1,118)_ = 336.71	2.310e^−36^	0.49 (large)	[0.35, 0.61]
RT L scale	Instructions x Time pressure	F_(1,118)_ = 10.80	1.335e^−03^	0.02 (small)	[0.00, 0.10]
MD-time L scale	Instructions x Time pressure	F_(1,118)_ = 6.66	1.111e^−02^	0.02 (small)	[0.00, 0.09]

With respect to magnitude, η_G_^2^ = 0.02 is considered indicative of a small effect, η_G_^2^ = 0.13 a medium effect, and η_G_^2^ = 0.26 a large effect^50^.

### Classification models

To consider the contribution of all dependent variables in a single statistical model and to further investigate the accuracy of mouse tracking parameters in detecting faking-good behaviours when responding to personality inventories, several classification models were built using machine learning (ML) techniques. ML approach is useful when the focus of the analysis is prediction instead of explanation^53^. Moreover, learning algorithms allow to find patterns in highly complex datasets: they can be effective also in the presence of complicated non-linear interactions^54^. Using ML approach, it is possible to build very complex models (e.g., considering a large number of variables), which are difficult to build with traditional statistical methods^55^. Furthermore, model evaluation techniques (e.g., k-fold cross validation) are intended to guarantee that the reported results are not overly optimistic. These machine learning models were implemented using the data mining software WEKA 3.9^56^. Model accuracy was evaluated using a 10-fold cross-validation procedure^57^, which consisted of repeatedly partitioning the original sample into a training set to train the model, and a validation set to evaluate it. The original sample of 120 participants who performed the task twice (honest vs. faking-good) was randomly partitioned into 10 equal-size subsamples, or folds (10 folds of 24 tasks). Of the 10 subsamples, data from a single subsample was retained as validation data for testing the model, and the remaining 9 subsamples were used to generate training data. This process was repeated 10 times, with each of the 10 folds used exactly once as validation data. The results of the 10 folds were then averaged to produce a single estimation of prediction accuracy. For each model, accuracy, recall, precision, F-measure, and ROC area were reported. Finally, the weight of each variable (predictor) was examined by measuring the correlation (r_pb_) between each variable and the outcome (honest vs. faking-good).

In a second phase, to test the generalization of the classifiers’ performance on completely new data, some of the models were tested on the additional sample of 120 participants who had been recruited as an out-of-sample evaluation group. Because the models had been built to fit the original data, it was important to test how they would fit new and unseen data^58^. The new group of participants (the test set) was collected after the models were built, so the subjects had never been seen by the ML classifiers.

As stated above, classification accuracy was evaluated using ML algorithms. Specifically, these algorithms investigated whether the results were stable across classifiers or whether they depended on specific model assumptions. In fact, the algorithms that were chosen were representative of different underlying classification strategies, including regression, trees, and Bayesian statistics (i.e., logistic^59^, support vector machine (SVM)^60^, naïve Bayes^61^, and random forest^62^ classifiers). For example, naïve Bayes is a probabilistic classifier inspired by the Bayes theorem. Naïve Bayes is a conditional probability model^51^: given a problem instance to be classified, represented by a vector x = (x_1_, …, x_n_) representing some n features (independent variables), it assigns to this instance probabilities p(C_k_ | x_1_, …, x_n_) for each of K possible outcomes or classes C_k_.

### ML model evaluation on all predictors

Using a 10-fold cross-validation procedure, the classification models were first built and evaluated on all predictors (i.e. L, K, S, and VR T-scores; L, K, S, and VR MD; L, K, S and VR tAUC; L, K, S, and VR RTs; L, K, S, and VR MD-time; L, K, S, and VR vel_x_; L, K, S, and VR vel_y_). The results (see Table 6) demonstrated stable classification accuracy across all classifiers, ranging from 76.25% to 80%, with the random forest classifier demonstrating the best performance (80%).

**Table 6:** Results from the ML models evaluated on the entire set of predictors. For each classifier, the following metrics obtained by the 10-fold cross-validation procedure are reported: accuracy, precision, recall, F-measure, and ROC area.

Classifier	Accuracy	Precision	Recall	F-measure	ROC area
Logistic	76.25%	0.763	0.763	0.762	0.825
SVM	78.33%	0.784	0.783	0.783	0.783
Naïve Bayes	76.67%	0.767	0.767	0.767	0.840
Random forest	80%	0.803	0.800	0.799	0.842

Analysis of the weight of predictors revealed that the variables most correlated with the outcome were the following: T-score S scale (r_pb_ = 0.587), T-score VR scale (r_pb_ = 0.574), T-score L scale (r_pb_ = 0.564), vel_x_ L scale (r_pb_ = 0.564), T-score K scale (r_pb_ = 0.545), vel_x_ S scale (r_pb_ = 0.528), vel_x_ K scale (r_pb_ = 0.519), and RT L scale (r_pb_ = 0.203). For all other variables, r_pb_ value was less than 0.2. Moreover, using logistic regression we calculated the AUC value of the ROC curve for each independent variable, obtaining the following results: T-score S scale AUC = 0.839, T-score L scale AUC = 0.824, T-score K scale AUC = 0.810, T-score VR scale AUC = 0.827, MD S scale AUC = 0.497, MD L scale AUC = 0.536, MD K scale AUC = 0.528, MD VR scale AUC = 0.483, tAUC S scale AUC = 0.510, tAUC L scale AUC = 0.527, tAUC K scale AUC = 0.514, tAUC VR scale AUC = 0.495, RT S scale AUC = 0.555, RT L scale AUC = 0.616, RT K scale AUC = 0.573, RT VR scale AUC = 0.497, MD-time S scale AUC = 0.517, MD-time L scale AUC = 0.583, MD-time K scale AUC = 0.553, MD-time VR scale AUC = 0.547, vel_x_ S scale AUC = 0.810, vel_x_ L scale AUC = 0.824, vel_x_ K scale AUC = 0.794, vel_x_ VR scale AUC = 0.573, vel_y_ S scale AUC = 0.522, vel_y_ L scale AUC = 0.529, vel_y_ K scale AUC = 0.564, vel_y_ VR scale AUC = 0.591. In other words, the major contribution to prediction accuracy came from the T-scores of all scales (S, L, K, and VR) and velocity along the x-axis for the S, L, and K scales.

### ML model evaluation on L scale predictors, only

As the univariate statistical analysis highlighted that honest and faking-good respondents mainly differed on temporal and spatial parameters on the L scale, only, a second set of ML models was evaluated using only L scale predictors: T-score L scale, RT L scale, MD-time L scale, vel_x_ L scale, vel_y_ L scale, MD L scale, and tAUC L scale. This was also the rationale for administering only the L scale items to the out-of-sample group.

The results are reported in Table 7, for both the 10-fold cross-validation and the test on the out-of-sample group. Classifiers showed accuracies ranging from 72.5% to 75.42% in the cross-validation, whereas they ranged from 78.75% to 81.67% in the out-of-sample group. These results demonstrate that: i) accuracies were stable across all classifiers; and ii) the models showed good generalization on completely new data, as the accuracies on the out-of-sample group outperformed those of the training models. With respect to the weight of predictors, T-score, vel_x_, and RT provided the most significant contributions to the model, while the others only fine-tuned the already good classification.

**Table 7:** Results from the ML models evaluated on the L scale predictors, only.

Classifier	Accuracy	Precision	Recall	F-measure	ROC area
**10-fold cross-validation**					
Logistic	74.58%	0.747	0.746	0.746	0.825
SVM	75.42%	0.761	0.754	0.753	0.754
Naïve Bayes	72.5%	0.728	0.725	0.724	0.820
Random Forest	75%	0.750	0.750	0.750	0.822
**Test on the out-of-sample**					
Logistic	78.75%	0.788	0.788	0.787	0.863
SVM	81.67%	0.817	0.817	0.817	0.817
Naïve Bayes	78.75%	0.795	0.788	0.786	0.871
Random Forest	80.42%	0.806	0.804	0.804	0.887

The table reports the performance (accuracy, precision, recall, F-measure, and ROC area) obtained by each classifier in the 10-fold cross-validation, which was run on the original sample of 120 participants, and the results obtained by testing the model on the out-of-sample group.

## Discussion

The main aim of the present research was to explore whether kinematic indicators could improve the detection of subjects implementing faking-good behaviour when answering personality inventories, with and without time pressure.

### T-scores on the MMPI-2 underreporting scales (L, K, S) and the PPI-R VR scale

The results supported the first hypothesis (H1), according to which T-scores on the underreporting scales (L, K, S) of the MMPI-2 and the VR scale of the PPI-R were expected to be higher in the faking-good condition compared to the honest condition. Indeed, on all scales, T-scores were significantly higher in the faking-good condition compared to the honest one. This finding is in line with the results of previous studies, indicating that fakers obtain high scores on MMPI overreporting scales^63^. In this sense, it is not a startling result, as it simply reflects the fact that the study instructions were correctly understood by participants: subjects instructed to fake good presented themselves in a more positive way by selecting the socially desirable alternative.

Moreover, contrary to expectations, the second hypothesis (H2), which expected T-scores on the selected underreporting scales to be higher in the faking-good speeded condition compared to the faking-good unspeeded condition and T-scores of honest respondents to not show any significant difference between the speeded and unspeeded conditions, found only partial support. Neither faking-good nor honest respondents, in fact, showed any statistically significant difference between the speeded and unspeeded conditions, in terms of T-scores. On the one hand, honest respondents remained honest under the speeded condition, indicating that they were not affected by time pressure; on the other hand, faking-good respondents in the speeded condition did not show the expected significant increase in T-scores. This result does not exactly agree with the findings of previous studies^17,22,23^, demonstrating that a speeded condition induces fakers to significantly improve their self-presentation (as demonstrated by increased T-scores) relative to an unspeeded condition, on both the MMPI-2-RF L-r and K-r scales^17^ and the MMPI-2 L and K scales^23^. The authors of these studies suggested that time pressure may limit respondents’ ability to consider the appropriateness of endorsing particularly virtuous items, and this may lead them to enhance their positive self-presentation and subsequently present less believable profiles. In this study, however, the T-scores of faking-good respondents were higher in the speeded condition than the unspeeded condition, on all scales—albeit not significantly. This lack of significance could be ascribed to the order in which subjects in the third experimental group (H-FG/S) completed the tests: since the administrated questionnaires were comprised of the same items, participants who first completed them honestly and then completed them under faking-good instructions may have been biased by a learning effect. Specifically, these respondents might have remembered the content of some items from the first administration, and this knowledge might have interfered with the effect of time pressure that has otherwise been observed in the literature. In other words, fakers take longer to respond because they must first identify the answer that could provide the most socially desirable image of themselves and choose this response over a true evaluation of their personality characteristics and mental functioning. Time is also necessary for a third evaluation—one that serves to estimate whether a particular answer will appear “too fake” and should subsequently be discarded for fear of discovery. Carrying out this triple evaluation—relating the questionnaire item to one’s own person, identifying the most socially desirable answer, and identifying whether the question might moderate faking-good behaviour—requires time. Temporal pressure reduces the available evaluation time and likely leads fakers to omit the last step of the decision process, making their faking behaviour more easily discovered. However, in the present study, faking-good respondents who already knew the item content because they had previously filled it in honestly (i.e., group 3, H-FG/S) may have been able to save sufficient time to carry out all three of the evaluation steps and therefore lie in a less detectable way. Other subjects, who knew the contents of the questions when filling out the questionnaire for a second time but were instructed to do so honestly (i.e., group 4, FG-H/S) would not have altered their answer: indeed, the response chosen (true vs. false/true vs. true enough vs. false enough vs. false) was used to calculate the T-scores for the scales.

### Differences in mouse movements and trajectories between honest respondents and fakers

In the present study, mouse dynamics were used for the first time to investigate faking-good behaviour with respect to the validity scales of two personality questionnaires (the MMPI-2 and PPI-R). In the literature, mouse dynamics have been shown to provide useful behavioural cues to identify deception^33,64^, and the technique has already been successfully applied to detect faking-bad respondents^5,47^. In the present research, only for the L scale the results were consistent with the findings reported in previous studies, which have shown that, compared to honest participants, fakers take more time to respond to stimuli^18^ and outline wider trajectories when selecting a response^5^, albeit with small observed effects. Only in relation to the L scale, indeed, the results supported the fourth hypothesis (H4); on this scale, mouse movements were slower in the faking-good condition relative to the honest condition, since faking-good respondents spent more time than honest respondents in responding. In fact, fakers showed significantly slower RTs and MD-times only on the L scale. Furthermore, only on the L scale, faking-good respondents showed wider mouse trajectories than honest respondents (see MD and tAUC parameters) with very small effects. The fifth hypothesis (H5), which expected faking-good respondents’ mouse trajectories to be wider than those of honest respondents, was only partially supported.

It is worth noting that the L scale demonstrated the most sensitivity in distinguishing between honest and faking-good respondents on the basis of both temporal and spatial parameters. This higher sensitivity may be due to the fact that this scale has particularly obvious items, with content pertaining to weaknesses and minor flaws that are observable in everyday life situations (e.g., “At times I feel like swearing,” “I do not always tell the truth”). Conversely, on the K scale, which is less transparent and focuses on more complex behaviours, honest respondents may require more time to choose the alternative that they feel best describes them. In the present study, the PPI-R underreporting scale VR was the least sensitive in differentiating honest from faking-good respondents. The lower number of significant scores in the VR statistics may be linked to a feature of the PPI-R test, itself: contrary to the MMPI-2, which offers dichotomous choices (TRUE vs. FALSE), the PPI-R offers four alternatives (TRUE, TRUE ENOUGH, FALSE ENOUGH, FALSE). In line with a previous study by Kiesler (1966), in which subjects selecting from four alternatives took longer than those selecting between two alternatives, the VR scale may have presented both honest and faking-good respondents with a more cognitively demanding decision-making process in both the speeded and unspeeded conditions. Future research should aim at uncovering the influence of the number of alternatives on test items by using exclusively underreporting scales offering four alternatives.

### Effect of time pressure on mouse movements and trajectories

Besides exploring the possibility of using mouse trajectories to detect faking-good behaviour, the present study also analysed the effect of time pressure on mouse movements. Results showed that participants in the speeded condition responded to all MMPI-2 items faster than those in the unspeeded condition (see RT and MD-time parameters). This result primarily reflects the fact that the instructions given to participants were effective in creating time pressure on subjects assigned to the speeded experimental condition, giving support for the third hypothesis (H3), which expected mouse movements to be faster in the speeded condition relative to the unspeeded condition. Furthermore, the result suggests that time pressure has an effect on the temporal parameters measured via mouse tracking that, as previously explained, are not necessarily the same as those measured by previous studies (which have typically used RTs). A more interesting finding is that time pressure affected the shape of the trajectories outlined by participants. Indeed, the statistical analysis found support for the sixth hypothesis (H6), which expected mouse trajectories for subjects in the speeded condition to be wider than those in the unspeeded condition. Specifically, MD was particularly sensitive to time pressure, with participants in the speeded condition showing greater MD than those in the unspeeded condition, even if with small effects. This result seems to corroborate the hypothesis that it is difficult to control more than one movement parameter, so the performance of participants decreases in spatial terms when time is limited.

Finally, as no significant interaction was found between the time pressure and instruction conditions, it can be concluded that time pressure is not a critical factor for the detection of faking-good behaviour when kinematic parameters are available.

### Predictive value of the technique

To investigate the predictive value of the abovementioned variables (T-scores and mouse dynamics) in detecting faking-good respondents in relation to the validity scales of personality questionnaires (i.e., the MMPI-2 L, K, and S scales and the PPI-2 VR scale), ML models were trained and validated. The results can be summarized as follows:

- The models achieved accuracies ranging from 72% to 80%.
- Different ML algorithms based on different classification strategies produced similar accuracies, confirming that the results were not dependent on the model assumptions.
- The most significant contribution to prediction accuracy was provided by T-scores and velocity along the x-axis; this was true for all scales, except for vel_x_ of the VR scale. RT of the L scale seemed to fine-tune the models.
- Entering only L scale predictors in the models produced similar results as entering all variables as predictors.
- The models that were built on the original sample performed with similar accuracy when tested on the out-of-sample, showing very good generalization to new data.

To conclude, this exploratory study suggests that some parameters of mouse dynamics—especially velocity on the x-axis—could be useful for detecting subjects who fake good when completing the validity scales of the MMPI-2 and PPI-R personality questionnaires, independent of whether a time pressure condition is imposed. However, upon comparing the accuracy performance obtained in this study (72–80%) with the accuracies reported in previous studies, it seems that mouse parameters may be less accurate than simple RT analysis (with reported accuracy ranging from 75–95%)^22^ for this task. The present findings are still preliminary and confirmatory studies are needed. Future research would benefit from studies situated in an ecological context; for example, studies with actual job applicants or child custody litigants. This would help to achieve generalizability with previous results obtained with instructed participants and experimental/manipulated designs, with the aim of including behavioural features for faking detection in personnel and forensic real-life settings^55^. Moreover, future studies could focus on improving converging validity by applying additional behavioural and implicit parameters and measuring these with eye-tracking^65^ and face-reading techniques^66^.

## Supplementary information

Supplementary Information.

## Data Availability

The dataset that was generated and analyzed for the current study is publicly available, along with the source code of the task, the analysis code, and the instructions: 10.5281/zenodo.3529450.

## References

[1] Anastasi, A. *Psychological testing*. (Macmillan Publishing Co, Inc., 1988).

[2] Ziegler, M., MacCann, C. & Roberts, R. D. In Ne*w p*erspect*iv*es on *faking in personality assessment* (eds. Ziegler, M., MacCann, C. & Roberts, R. D.) 3–16 (Oxford University Press, 2012).

[3] Griffith, R. L. & Converse, P. D. In N*ew* perspect*iv*es on *faking in personality assessment* (eds. Ziegler, M., MacCann, C. & Roberts, R. D.) 34–52 (Oxford University Press, 2012).

[4] Bass C, Halligan PW (2007). Illness related deception: social or psychiatric problem?. J. R. Soc. Med..

[5] Monaro, M. *et al*. The Detection of Malingering: A New Tool to Identify Made-Up Depression. *Front. Psychiatry* **9**, (2018). 10.3389/fpsyt.2018.00249 PMC6002526 29937740

[6] Rogers, R., Sewell, K. W. & Gillard, N. D. *Structured Interview of Reported Symptons*. (Psychological Assessment Resources, 2010).

[7] Smith GP, Burger GK (1997). Detection of malingering: validation of the Structured Inventory of Malingered Symptomatology (SIMS). J. Am. Acad. Psychiatry Law.

[8] Viglione DJ, Giromini L, Landis P (2017). The Development of the Inventory of Problems–29: A Brief Self-Administered Measure for Discriminating Bona Fide From Feigned Psychiatric and Cognitive Complaints. J. Pers. Assess..

[9] Mazza C (2019). Indicators to distinguish symptom accentuators from symptom producers in individuals with a diagnosed adjustment disorder: A pilot study on inconsistency subtypes using SIMS and MMPI-2-RF. PLoS One.

[10] Roma Paolo, Giromini Luciano, Burla Franco, Ferracuti Stefano, Viglione Donald J., Mazza Cristina (2019). Ecological Validity of the Inventory of Problems-29 (IOP-29): an Italian Study of Court-Ordered, Psychological Injury Evaluations Using the Structured Inventory of Malingered Symptomatology (SIMS) as Criterion Variable. Psychological Injury and Law.

[11] Paulhus, D. L. In *The r*ole of *co*nstru*cts in psychological and educational measurement* (eds. Braun, H. I., Jackson, D. N. & Wiley, D. E.) 49–69 (Lawrence Erlbaum Associates Publishers, 2002).

[12] Sartori Giuseppe, Zangrossi Andrea, Monaro Merylin (2018). Deception Detection With Behavioral Methods. Detecting Concealed Information and Deception.

[13] Holden RR, Kroner DG, Fekken GC, Popham SM (1992). A model of personality test item response dissimulation. J. Pers. Soc. Psychol..

[14] Maricuţoiu LP, Sârbescu P (2016). The relationship between faking and response latencies: a meta-analysis. Eur. J. Psychol. Assess..

[15] Holden RR, Kroner DG (1992). Relative efficacy of differential response latencies for detecting faking on a self-report measure of psychopathology. Psychol. Assess..

[16] Foerster, A., Pfister, R., Schmidts, C., Dignath, D. & Kunde, W. Honesty saves time (and justifications). *Front. Psychol*. **4**, (2013). 10.3389/fpsyg.2013.00473 PMC3719030 23888151

[17] Roma, P. *et al*. Could Time Detect a Faking-Good Attitude? A Study With the MMPI-2-RF. *Front. Psychol*. **9**, (2018). 10.3389/fpsyg.2018.01064 PMC6069678 30090076

[18] Monaro, M., Gamberini, L., Zecchinato, F. & Sartori, G. False Identity Detection Using Complex Sentences. *Front. Psychol*. **9**, (2018). 10.3389/fpsyg.2018.00283 PMC5845552 29559945

[19] Vasilopoulos NL, Reilly RR, Leaman JA (2000). The influence of job familiarity and impression management on self-report measure scale scores and response latencies. J. Appl. Psychol..

[20] Shalvi S, Eldar O, Bereby-Meyer Y (2012). Honesty Requires Time (and Lack of Justifications). Psychol. Sci..

[21] Khorramdel L, Kubinger KD (2006). The effect of speediness on personality questionnaires: an experiment on applicants within a job recruiting procedure. Psychol. Sci..

[22] Mazza, C. *et al*. Introducing Machine Learning to Detect Personality Faking-Good in a Male Sample: A New Model Based on Minnesota Multiphasic Personality Inventory-2 Restructured Form Scales and Reaction Times. *Front. Psychiatry* **10**, (2019). 10.3389/fpsyt.2019.00389 PMC6593269 31275176

[23] Roma, P. *et al*. Faking-Good Behavior in Self-Favorable Scales of the MMPI-2. *Eur. J. Psychol. Assess*. 1–9. 10.1027/1015-5759/a000511 (2019).

[24] Verschuere B, Köbis NC, Bereby-Meyer Y, Rand D, Shalvi S (2018). Taxing the Brain to Uncover Lying? Meta-analyzing the Effect of Imposing Cognitive Load on the Reaction-Time Costs of Lying. J. Appl. Res. Mem. Cogn..

[25] Freeman JB, Ambady N (2010). MouseTracker: software for studying real-time mouse-tracking method. Behav. Res. Methods.

[26] Dale R, Kehoe C, Spivey MJ (2007). Graded motor responses in the time course of categorizing atypical exemplars. Mem. Cognit..

[27] Freeman JB, Ambady N, Rule NO, Johnson KL (2008). Will a category cue attract you? Motor output reveals dynamic competition across person construal. J. Exp. Psychol. Gen..

[28] Freeman, J. B., Dale, R. & Farmer, T. A. Hand in motion reveals mind in motion. *Front. Psychol*. **2**, (2011). 10.3389/fpsyg.2011.00059 PMC3110497 21687437

[29] McKinstry C, Dale R, Spivey MJ (2008). Action Dynamics Reveal Parallel Competition in Decision Making. Psychol. Sci..

[30] Song JH, Nakayama K (2008). Target selection in visual search as revealed by movement trajectories. Vision Res..

[31] Spivey MJ, Grosjean M, Knoblich G (2005). From The Cover: Continuous attraction toward phonological competitors. Proc. Natl. Acad. Sci..

[32] Berkman ET, Hutcherson CA, Livingston JL, Kahn LE, Inzlicht M (2017). Self-Control as Value-Based Choice. Curr. Dir. Psychol. Sci..

[33] Monaro M, Gamberini L, Sartori G (2017). The detection of faked identity using unexpected questions and mouse dynamics. PLoS One.

[34] Monaro Merylin, Fugazza Francesca Ileana, Gamberini Luciano, Sartori Giuseppe (2017). How Human-Mouse Interaction can Accurately Detect Faked Responses About Identity. Symbiotic Interaction.

[35] Monaro, M., Gamberini, L. & Sartori, G. Spotting faked identities via mouse dynamics using complex questions. in *Proceedings of the 32nd International BCS Human Computer Interaction Conference (HCI 2018)*10.14236/ewic/HCI2018.8 (2018).

[36] Magnusson, K. Understanding Statistical Power and Significance Testing. Available at: https://rpsychologist.com/d3/NHST/. (Accessed: 30th October 2019).

[37] Butcher, J. N., Dahlstrom, W. G., Graham, J. R., Tellegen, A. & Kaemmer, B. *Manual for restandardized Minnesota Multiphasic Personality Inventory: MMPI-2. An interpretative and administrative guide*. (1989).

[38] Otto RK (2002). Use of the MMPI-2 in Forensic Settings. J. Forensic Psychol. Pract..

[39] Roma P, Pazzelli F, Pompili M, Girardi P, Ferracuti S (2013). Shibari: Double Hanging During Consensual Sexual Asphyxia. Arch. Sex. Behav..

[40] Roma P (2014). MMPI-2 in Child Custody Litigation. Eur. J. Psychol. Assess..

[41] Roma P, Piccinni E, Ferracuti S (2016). Using MMPI-2 in forensic assessment. Rass. Ital. di Criminol..

[42] Mazza, C. *et al*. MMPI-2-RF Profiles in Child Custody Litigants. *Front. Psychiatry* **10**, (2019). 10.3389/fpsyt.2019.00725 PMC6805769 31681037

[43] Pancheri, P. & Sirigatti, S. *MMPI-2 - Minnesota Multiphasic Personality Inventory − 2. Manuale*. (Giunti O.S. Organizzazioni Speciali, 1995).

[44] Sirigatti, S. & Stefanile, C. *MMPI-2: Aggiornamento all’adattamento italiano. Scale di validità, Harris-Lingoes, supplementari, di contenuto e PSY-5*. (Giunti O.S. Organizzazioni Speciali, 2011).

[45] Lilienfeld, S. O. & Widows, M. R. *Psychopathic Personality Inventory-Revised: professional manual*. (Psychological Assessment Resources, 2005).

[46] La Marca, S., Berto, D. & Rovetto, F. *Traduzione ed adattamento italiano del PPI-R: Psychopathic Personality Inventory Revised-PPI-R*. (Giunti O.S. Organizzazioni Speciali, 2008).

[47] Zago, S. *et al*. The Detection of Malingered Amnesia: An Approach Involving Multiple Strategies in a Mock Crime. *Front. Psychiatry* **10**, (2019). 10.3389/fpsyt.2019.00424 PMC6589901 31263432

[48] Mortensen EL, Gade A (1992). Linear versus normalized T scores as standardized neuropsychological test scores. Scand. J. Psychol..

[49] Colligan RC, Osborne D, Offord KP (1980). Linear transformation and the interpretation of MMPI T scores. J. Clin. Psychol..

[50] Cohen Jacob (2013). Statistical Power Analysis for the Behavioral Sciences.

[51] The R Project for statistical computing. Available at: https://www.r-project.org/. (Accessed: 10th October 2017) (2015).

[52] Shaffer JP (1995). Multiple Hypothesis Testing. Annu. Rev. Psychol..

[53] Yarkoni T, Westfall J (2017). Choosing Prediction Over Explanation in Psychology: Lessons From Machine Learning. Perspect. Psychol. Sci..

[54] Orrù, G., Monaro, M., Conversano, C., Gemignani, A. & Sartori, G. Machine Learning in Psychometrics and Psychological Research. *Front. Psychol*. **10**, (2020). 10.3389/fpsyg.2019.02970 PMC6966768 31998200

[55] Burla, F. *et al*. Use of the Parents Preference Test in Child Custody Evaluations: Preliminary Development of Conforming Parenting Index. *Mediterr. J. Clin. Psychol*. **7**, (2019).

[56] Hall MA (2009). The WEKA data mining software: an update. ACM SIGKDD Explor. Newsl..

[57] Kohavi, R. A study of cross-validation and bootstrap for accuracy estimation and model selection. In *Proceedings of the 14th International Joint Conference on Artificial Intelligence* **2**, 1137–1143 (Morgan Kaufmann, 1995).

[58] Dwork C (2015). The reusable holdout: preserving validity in adaptive data analysis. Science (80-.)..

[59] le Cessie, S. & van Houwelingen, J. C. Ridge estimators in logistic regression. *Appl. Stat.* **41**, 191–201 (1992).

[60] Keerthi, S. S., Shevade, S. K., Bhattacharyya, C. & Murthy, K. R. K. Improvements to platt’s SMO algorithm for SVM classifier design. *Neural Comput.* **13**, 637–649 (2001).

[61] John, G. H. & Langley, P. Estimating continuous distributions in Bayesian classifiers. in Proceeding of the 11th Conference on Uncertainty in Artificial Intelligence. 338–345 (1995).

[62] Breiman, L. Random forest. *Mach. Learn.* **45**, 5–32 (2001).

[63] Roma P (2019). Drinking and driving relapse: Data from BAC and MMPI-2. PLoS One.

[64] Duran ND, Dale R, McNamara DS (2010). The action dynamics of overcoming the truth. Psychon. Bull. Rev..

[65] van Hooft EAJ, Born MP (2012). Intentional response distortion on personality tests: Using eye-tracking to understand response processes when faking. J. Appl. Psychol..

[66] Liem Cynthia C. S., Langer Markus, Demetriou Andrew, Hiemstra Annemarie M. F., Sukma Wicaksana Achmadnoer, Born Marise Ph., König Cornelius J. (2018). Psychology Meets Machine Learning: Interdisciplinary Perspectives on Algorithmic Job Candidate Screening. The Springer Series on Challenges in Machine Learning.

