# Supplementary material for: Use of mouse-tracking software to detect faking-good behavior on personality questionnaires: an explorative study
Source: Sci Rep. 2020 Mar 16;10:4835. doi: 10.1038/s41598-020-61636-5 (PMC7075885; doi:10.1038/s41598-020-61636-5)
Supplement: Supplementary file 1 — Supplementary Information. [file 41598_2020_61636_MOESM1_ESM.docx]

**Use of mouse-tracking software to detect faking-good behavior on personality questionnaires: an explorative study**

**Cristina Mazza^1*^, Merylin Monaro^2*^, Franco Burla^1^, Marco Colasanti^1^, Graziella Orrù^3^, Stefano Ferracuti^1^, Paolo Roma^1†^**

^1^ Department of Human Neuroscience, Sapienza University of Rome, Rome, Italy

^2^ Department of General Psychology, University of Padova, Padova, Italy

^3^ Department of Surgical, Medical, Molecular & Critical Area Pathology, University of Pisa, Pisa, Italy

*Authors contributed to the paper equally

† Correspondence: paolo.roma@uniroma1.it

**Supplementary Information**

**Table S1.** Items (identified by number) comprising the selected MMPI-2 (L, K, S) and PPI-R (VR) scales.

|  | **N° Item** |
| --- | --- |
| **L scale** | 16, 29, 41, 51, 77, 93, 102, 107, 123, 139, 153, 183, 203, 232, 260 |
| **K scale** | 29, 37, 58, 76, 83, 110, 116, 122, 127, 130, 136, 148, 157, 158, 167, 171, 196, 213, 243, 267, 284, 290, 330, 338, 339, 341, 346, 348, 356, 365 |
| **S scale** | 15, 50, 58, 76, 81, 87, 89, 104, 110, 120, 121, 123, 148, 154, 184, 194, 196, 205, 213, 225, 264, 279, 284, 290, 302, 337, 341, 346, 352, 373, 374, 403, 420, 423, 428, 430, 433, 442, 445, 449, 461, 486, 487, 523, 534, 538, 542, 545, 547, 560 |
| **VR scale** | 20, 37, 42, 59, 64, 81, 86, 95, 106, 117, 128, 139, 150 |

**Table S2.** Table lists and description of the variables derived from the experimental task.

| **Variable** | **Description** |
| --- | --- |
| L T-score | T-score obtained by the subject on the L scale of the MMPI-2 |
| K T-score | T-score obtained by the subject on the K scale of the MMPI-2 |
| S T-score | T-score obtained by the subject on the S scale of the MMPI-2 |
| VR T-score | T-score obtained by the subject on the VR scale of the PPI-R |
| L RT | Average reaction time to the questions of the MMPI-2 L scale |
| K RT | Average reaction time to the questions of the MMPI-2 K scale |
| S RT | Average reaction time to the questions of the MMPI-2 S scale |
| VR RT | Average reaction time to the questions of the PPI-R VR scale |
| L MD-time | Average MD-time to the questions of the MMPI-2 L scale |
| K MD-time | Average MD-time to the questions of the MMPI-2 K scale |
| S MD-time | Average MD-time to the questions of the MMPI-2 S scale |
| VR MD-time | Average MD-time to the questions of the PPI-R VR scale |
| L velx | Average velocity on the x-axis for questions on the MMPI-2 L scale |
| K velx | Average velocity on the x-axis for questions on the MMPI-2 K scale |
| S velx | Average velocity on the x-axis for questions on the MMPI-2 S scale |
| VR velx | Average velocity on the x-axis for questions on the PPI-R VR scale |
| L vely | Average velocity on the y-axis for questions on the MMPI-2 L scale |
| K vely | Average velocity on the y-axis for questions on the MMPI-2 K scale |
| S vely | Average velocity on the y-axis fore questions on the MMPI-2 S scale |
| VR vely | Average velocity on the y-axis for questions on the PPI-R VR scale |
| L MD | Average maximum deviation for questions on the MMPI-2 L scale |
| K MD | Average maximum deviation for questions on the MMPI-2 K scale |
| S MD | Average maximum deviation for questions on the MMPI-2 S scale |
| VR MD | Average maximum deviation for questions on the PPI-R VR scale |
| L AUC | Average area under the curve for questions on the MMPI-2 L scale |
| K AUC | Average area under the curve for questions on the MMPI-2 K scale |
| S AUC | Average area under the curve for questions on the MMPI-2 S scale |
| VR AUC | Average area under the curve for questions on the PPI-R VR scale |

**Table S3.** Means and standard deviations for all dependent variables.

|  | **H-U**  **M (SD)** | **H-S**  **M (SD)** | **FG-U**  **M (SD)** | **FG-S**  **M (SD)** |
| --- | --- | --- | --- | --- |
| **T-score S** | 43.97 (8.90) | 43.17 (8.94) | 57.73 (9.48) | 57.23 (11.16) |
| **T-score K** | 45.37 (8.83) | 45.53 (8.31) | 56.12 (8.10) | 57.13 (9.35) |
| **T-score L** | 47.42 (8.75) | 47.38 (9.66) | 61.3 (11.18) | 64.55 (14.98) |
| **T-score VR** | 49.08 (10.56) | 50.45 (11.96) | 66.37 (12.53) | 67.25 (13.72) |
| **RT S** | 5232.61 (2157.09) | 4220.91 (1092.72) | 5404.79 (1547.49) | 4519.71 (1075.51) |
| **RT K** | 4928.55 (2012.77) | 3942.87 (913.36) | 5164.71 (1486.47) | 4249.18 (965.72) |
| **RT L** | 4210.18 (1362.99) | 3469.27 (867.24) | 4858.60 (1542.59) | 3871.92 (895.39) |
| **RT VR** | 5299.23 (1623.13) | 4629.13 (1147.81) | 5484.85 (1794.82) | 4747.49 (1126.51) |
| **MD-time S** | 3424.64 (1273.58) | 2748.15 (764.31) | 3519.65 (1131.96) | 2921.27 (792.67) |
| **MD-time K** | 3231.04 (1256.33) | 2548.78 (643.16) | 3377.72 (996.80) | 2744.44 (716.72) |
| **MD-time L** | 2699.57 (874.07) | 2199.59 (634.75) | 2994.81 (977.95) | 2471.85 (744.63) |
| **MD-time VR** | 3300.22 (1262.90) | 2943.45 (672.64) | 3399.30 (1243.55) | 3099.80 (739.08) |
| **velx S** | -0.00112 (0.00216) | -0.00118 (0.00212) | 0.00141 (0.00199) | 0.00147 (0.00214) |
| **velx K** | -0.0012 (0.00209) | -0.00119 (0.00202) | 0.00111 (0.00187) | 0.00139 (0.00214) |
| **velx L** | -0.00312 (0.00235) | -0.00314 (0.00254) | 0.00060 (0.00298) | 0.00137 (0.00398) |
| **velx VR** | 0.00066 (0.00106) | 0.00056 (0.00134) | 0.00046 (0.00102) | 0.00021 (0.00112) |
| **vely S** | 0.01293 (0.00016) | 0.01295 (0.00014) | 0.01291 (0.00015) | 0.01292 (0.00012) |
| **vely K** | 0.01292 (0.00019) | 0.01296 (0.00016) | 0.01289 (0.00014) | 0.01292 (0.00013) |
| **vely L** | 0.01294 (0.00017) | 0.01293 (0.00015) | 0.01290 (0.00017) | 0.01293 (0.00016) |
| **vely VR** | 0.00007 (0.00098) | 0.00023 (0.00129) | 0.00036 (0.00114) | 0.00060 (0.00099) |
| **MD S** | 0.31177 (0.17194) | 0.40055 (0.18696) | 0.34087 (0.18223) | 0.41087 (0.20546) |
| **MD K** | 0.31322 (0.18150) | 0.38201 (0.17882) | 0.34330 (0.19792) | 0.41545 (0.20627) |
| **MD L** | 0.27230 (0.16478) | 0.32882 (0.17425) | 0.28685 (0.17126) | 0.39740 (0.20698) |
| **MD VR** | 0.19178 (0.08835) | 0.17251 (0.08959) | 0.19669 (0.10473) | 0.18644 (0.09493) |
| **AUC S** | 0.64738 (0.46002) | 0.87811 (0.57280) | 0.75942 (0.62904) | 0.90349 (0.62732) |
| **AUC K** | 0.66746 (0.53361) | 0.80776 (0.51426) | 0.74209 (0.64175) | 0.88189 (0.61436) |
| **AUC L** | 0.54235 (0.48167) | 0.65858 (0.45206) | 0.62026 (0.67700) | 0.81168 (0.55784) |
| **AUC VR** | 1.83185 (2.99841) | 0.96968 (1.46761) | 2.36537 (8.19277) | 1.56024 (2.87678) |
| **x-flip S** | 5.40 (1.62) | 6.11 (1.33) | 5.45 (1.59) | 5.96 (1.33) |
| **x-flip K** | 5.47 (1.52) | 6.06 (1.32) | 5.65 (1.45) | 6.22 (1.40) |
| **x-flip L** | 5.73 (1.85) | 6.26 (1.39) | 5.80 (1.63) | 6.24 (1.42) |
| **x-flip VR** | 5.50 (2.05) | 5.96 (1.81) | 5.69 (2.32) | 6.26 (1.83) |
| **y-flip S** | 4.73 (1.58) | 5.57 (1.56) | 4.84 (1.52) | 5.30 (1.44) |
| **y-flip K** | 4.81 (1.63) | 5.51 (1.52) | 4.94 (1.49) | 5.54 (1.56) |
| **y-flip L** | 4.89 (1.75) | 5.47 (1.55) | 5.05 (1.66) | 5.52 (1.67) |
| **y-flip VR** | 5.33 (2.20) | 5.85 (2.34) | 4.91 (2.16) | 5.63 (2.30) |

**Table S4.** Confidence Intervals (95% CI) for non-significant results from the ANOVA mixed models computed on the T-scores of the L, K, and S scales of the MMPI-2 and the VR scale of the PPI-R.

| **T-score variable** | **Effect** | **95% CI** |
| --- | --- | --- |
| T-score S scale | Time pressure | [0.00, 0.04] |
| T-score K scale | Time pressure | [0.00, 0.04] |
| T-score L scale | Time pressure | [0.00, 0.06] |
| T-score VR scale | Time pressure | [0.00, 0.05] |
| T-score S scale | Instructions x Time pressure | [0.00, 0.02] |
| T score K scale | Instructions x Time pressure | [0.00, 0.04] |
| T-score L scale | Instructions x Time pressure | [0.00, 0.06] |
| T-score VR scale | Instructions x Time pressure | [0.00, 0.02] |

**Table S5.** Confidence Intervals (95% CI) for non-significant results from the ANOVA mixed models computed on RT, MD-time, vel_x_, and vel_y_ for each scale (L, K, S, VR).

| **Temporal variable** | **Effect** | **95% CI** |
| --- | --- | --- |
| MD-time VR scale | Time pressure | [0.00, 0.09] |
| RT S scale | Instructions | [0.00, 0.06] |
| RT K scale | Instructions | [0.00, 0.07] |
| RT VR scale | Instructions | [0.00, 0.05] |
| MD-time S scale | Instructions | [0.00, 0.06] |
| MD-time K scale | Instructions | [0.00, 0.07] |
| MD-time VR scale | Instructions | [0.00, 0.06] |
| vely S scale | Instructions | [0.00, 0.06] |
| vely K scale | Instructions | [0.00, 0.08] |
| velx VR scale | Instructions | [0.00, 0.09] |
| RT S scale | Instructions x Time pressure | [0.00, 0.03] |
| RT K scale | Instructions x Time pressure | [0.00, 0.02] |
| RT L scale | Instructions x Time pressure | [0.00, 0.05] |
| RT VR scale | Instructions x Time pressure | [0.00, 0.02] |
| MD-time S scale | Instructions x Time pressure | [0.00, 0.03] |
| MD-time K scale | Instructions x Time pressure | [0.00, 0.03] |
| MD-time L scale | Instructions x Time pressure | [0.00, 0.01] |
| MD-time VR scale | Instructions x Time pressure | [0.00, 0.03] |
| velx S scale | Instructions x Time pressure | [0.00, 0.03] |
| velx K scale | Instructions x Time pressure | [0.00, 0.04] |
| velx L scale | Instructions x Time pressure | [0.00, 0.06] |
| velx VR scale | Instructions x Time pressure | [0.00, 0.04] |
| vely S scale | Instructions x Time pressure | [0.00, 0.03] |
| vely K scale | Instructions x Time pressure | [0.00, 0.03] |
| vely L scale | Instructions x Time pressure | [0.00, 0.06] |
| vely VR scale | Instructions x Time pressure | [0.00, 0.03] |

| **Spatial variable** | **Effect** | **95% CI** |
| --- | --- | --- |
| MD VR scale | Time pressure | [0.00, 0.06] |
| tAUC S scale | Time pressure | [0.00, 0.09] |
| tAUC K scale | Time pressure | [0.00, 0.07] |
| tAUC L scale | Time pressure | [0.00, 0.08] |
| tAUC VR scale | Time pressure | [0.00, 0.06] |
| MD S scale | Instructions | [0.00, 0.05] |
| MD K scale | Instructions | [0.00, 0.07] |
| MD VR scale | Instructions | [0.00, 0.05] |
| tAUC S scale | Instructions | [0.00, 0.06] |
| tAUC K scale | Instructions | [0.00, 0.06] |
| tAUC VR scale | Instructions | [0.00, 0.06] |
| MD S scale | Instructions x Time pressure | [0.00, 0.04] |
| MD K scale | Instructions x Time pressure | [0.00, 0.01] |
| MD L scale | Instructions x Time pressure | [0.00, 0.06] |
| MD VR scale | Instructions x Time pressure | [0.00, 0.04] |
| tAUC S scale | Instructions x Time pressure | [0.00, 0.04] |
| tAUC K scale | Instructions x Time pressure | [0.00, 0.99] |
| tAUC L scale | Instructions x Time pressure | [0.00, 0.04] |
| tAUC VR scale | Instructions x Time pressure | [0.00, 0.00] |

**Table S6.** Confidence Intervals (95% CI) for non-significant results from the ANOVA mixed models computed on MD and tAUC for each scale (L, K, S, VR).

**Table S7.** Confidence Intervals (95% CI) for non-significant results from the ANOVA mixed models computed on the T-scores, RT, MD-time, vel_x_, vel_y_, MD and tAUC of the L scale of the MMPI-2 for the 120 additional volunteers recruited as an out-of-sample evaluation group.

| **L scale variables** | **Effect** | **95% CI** |
| --- | --- | --- |
| T-score L scale | Time pressure | [0.00, 0.00] |
| t-AUC L scale | Time pressure | [0.00, 0.08] |
| velx L scale | Time pressure | [0.00, 0.99] |
| vely L scale | Time pressure | [0.00, 0.04] |
| vely L scale | Instructions | [0.00, 0.06] |
| T-score L scale | Instructions x Time pressure | [0.00, 0.03] |
| MD L scale | Instructions x Time pressure | [0.00, 0.05] |
| tAUC L scale | Instructions x Time pressure | [0.00, 0.05] |
| velx L scale | Instructions x Time pressure | [0.00, 0.03] |
| vely L scale | Instructions x Time pressure | [0.00, 0.03] |
